# Regulation of Bicarbonate Secretion in Marine Fish Intestine by the Calcium-Sensing Receptor

**DOI:** 10.3390/ijms19041072

**Published:** 2018-04-04

**Authors:** Sílvia F. Gregório, Juan Fuentes

**Affiliations:** Centre of Marine Sciences (CCMar), Universidade do Algarve, Campus de Gambelas, 8005-139 Faro, Portugal; sfgregorio@ualg.pt

**Keywords:** sea bream, anterior intestine, bicarbonate secretion and calcium sensing receptor

## Abstract

In marine fish, high epithelial intestinal HCO_3_^−^ secretion generates luminal carbonate precipitates of divalent cations that play a key role in water and ion homeostasis. The present study was designed to expose the putative role for calcium and the calcium-sensing receptor (CaSR) in the regulation of HCO_3_^−^ secretion in the intestine of the sea bream (*Sparus aurata* L.). Effects on the expression of the CaSR in the intestine were evaluated by qPCR and an increase was observed in the anterior intestine in fed fish compared with unfed fish and with different regions of intestine. CaSR expression reflected intestinal fluid calcium concentration. In addition, anterior intestine tissue was mounted in Ussing chambers to test the putative regulation of HCO_3_^−^ secretion in vitro using the anterior intestine. HCO_3_^−^ secretion was sensitive to varying calcium levels in luminal saline and to calcimimetic compounds known to activate/block the CaSR i.e., R 568 and NPS-2143. Subsequent experiments were performed in intestinal sacs to measure water absorption and the sensitivity of water absorption to varying luminal levels of calcium and calcimimetics were exposed as well. It appears, that CaSR mediates HCO_3_^−^ secretion and water absorption in marine fish as shown by responsiveness to calcium levels and calcimimetic compounds.

## 1. Introduction

The contribution of the intestine to total calcium uptake is an important factor derived from the high drinking rates of seawater fish and dietary intake. Drinking is necessary to compensate osmotic water losses [1,2,3], therefore water replacement by drinking becomes essential to sustain osmotic regulation [4]. Several studies have reported the involvement of endocrine and environmental factors in the regulation of the amount of water ingestion by seawater fish [4,5,6,7,8,9,10].

Marine fish drink large amounts of seawater and processing of ingested water to compensate branchial osmotic water loss is relevant for osmoregulation [5,6,11]. The ingested water needs to be processed to facilitate absorption. The first step of processing takes places in the oesophagus were seawater is rapidly desalted by NaCl absorption [12,13]. In the intestine, water absorption is primarily associated with NaCl cotransport; and over the length of the entire gastrointestinal tract approximately 99% of NaCl is absorbed and excreted [1,13,14,15,16,17,18,19]. However, recent reports [20] have demonstrated that Cl^−^ uptake linked to HCO_3_^−^ via the Cl^−^/HCO_3_^−^ exchanger is relevant to water uptake. Members of the Slc26 family are responsible for this apical mechanism in fish enterocytes, more specifically the SLC26a6 as demonstrated for pufferfish (*Takifugu obscurus*) and toadfish (*Opsanus beta*) [21,22]. HCO_3_^−^ enters the cell through a basolateral Na^+^/HCO_3_^−^ co-transporter belonging to the Slc4 family or is formed by the intracellular action of carbonic anhydrases that hydrate the CO_2_ resulting in the production of HCO_3_^−^ and H^+^ ions reviewed by [20]. Several studies have demonstrated that intestinal carbonate precipitate formation has an important role in osmoregulation in marine fish. Luminal HCO_3_^−^ concentrations and the associated alkaline conditions in the intestinal fluids result in CaCO_3_ precipitation [18,19,23,24]. The resulting precipitation of CaCO_3_ facilitates water absorption [18,19,24], by lowering the luminal fluid osmotic pressure, as well as facilitating Ca^2+^ homeostasis [5,25] by reducing excess Ca^2+^ entry into the body. The digestive tract of fish has a dual role as a food-processing organ and as an osmoregulatory organ [26,27]. However, little attention has been devoted to dietary source of additional calcium or as a regulatory factor. Calcium availability may constitute by itself a fundamental factor to condition intestinal function in relation to calcium transport, but also to HCO_3_^−^ regulation, due to the prevailing acid digestion of most fish [23,28,29].

Several hormones, such as stanniocalcin (STC), calcitonin, somatolactin (SL), parathyroid hormone-related protein (PTHrP) and, parathyroid hormone (PTH) are linked in the control of calcium transport in fish [5,7,30,31,32,33,34]. And recently, in the sea bream [10,35] it was demonstrated that the internal modulation of epithelial HCO_3_^−^ secretion and the associated carbonate precipitation are under endocrine control highlighting a novel physiological role for calcium regulation.

Following this line of thinking, the literature tells the story that calcium-sensing receptor (CaSR) may have evolved in the marine environment to support osmo-regulation [36]. The CaSR is expressed in endocrine tissues that secrete calciotropic and other hormones such as the pituitary gland and the corpuscles of Stannius [32,36,37] and may play a central role in global integrative signalling. In addition the CaSR is expressed in ion-transporting tissues, such as the kidney, intestine, gills, and the elasmobranch rectal gland [36,38,39,40,41,42].

Limited information is available on the molecular and pharmacological characterization of the calcium sensing receptor in fish. The calcimimetics are synthetic small organic compounds that bind to the transmembrane region of the CaSR and act as positive/negative allosteric modulators, and have been characterised in terrestrial vertebrates, especially in humans [43,44,45] but not in fish. Among the pharmacological regulators of CaSR agonists (e.g., R 568) and antagonists (e.g., NPS-2143) have been characterised. The CaSR agonists, R 568 [32,44], and antagonist, NPS-2143 [45,46], stimulate and inhibits CaSR activity, respectively. 

The objective of this study was to establish a putative physiological role for calcium and the CaSR in the regulation of HCO_3_^−^ secretion and water absorption in the intestine of the sea bream (*Sparus aurata* L.) and the results here presented provide the first solid evidence for a role of the CaSR in intestinal physiology in fish.

## 2. Results

### 2.1. CaSR Expression

**Figure 1:**
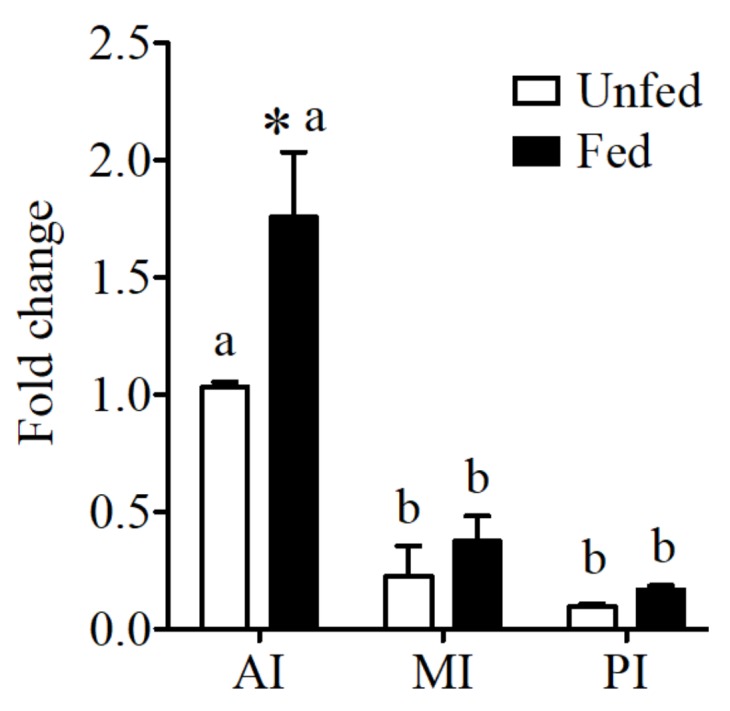
CaSR expression in anterior intestine (AI), mid intestine (MI), and rectum of the sea bream intestine in fed and unfed conditions. CaSR expression was normalized by 18S and expression levels are presented as fold-change from anterior intestine of unfed fish. The results are shown as mean ± SE (*n* = 5–7); differences among groups were evaluated by two-way ANOVA (followed by a Bonferroni post-hoc test), with feeding conditions and intestinal segment as independent variables within feeding groups different letters represent significantly different levels, between the same regions, * represents significant effects *p* < 0.05.

Expression of the CaSR in the intestine of the sea bream showed significantly higher levels in anterior intestine when compared to the other intestinal regions (Figure 1). In addition significant increases in expression were observed in the anterior intestine in fed fish in relation to unfed conditions. 

### 2.2. Effects of Feeding in Plasma and Fluid Calcium

**Figure 2:**
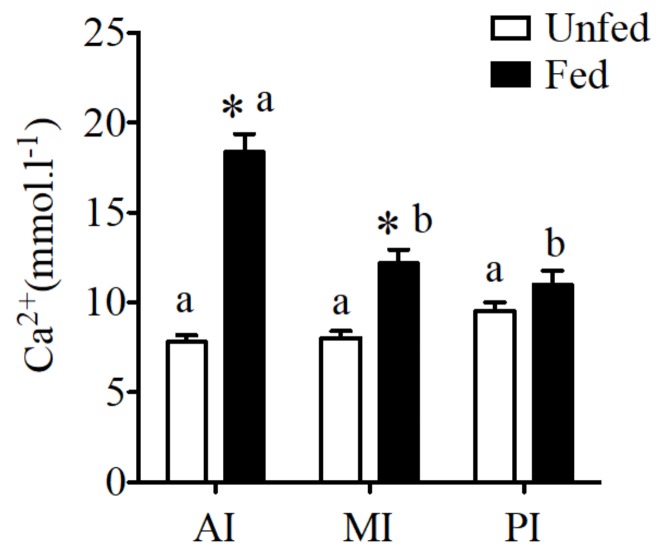
Regional calcium concentration (mmol·L^−1^) in anterior intestine (AI), mid intestine (MI), and rectum in fluids of sea bream collected from unfed and fed fish sampled 6 h after feeding. Results are shown as mean *±* SE (*n* = 8–10); differences among groups were evaluated by two-way ANOVA (followed by a Bonferroni post-hoc test), with feeding conditions and intestinal segment as independent variables within feeding groups different letters represent significantly different levels, between the same regions, * represents significant effects *p* < 0.05.

No difference was observed in fluid calcium concentration of unfed sea bream in different regions of the intestine. However, a significant increase of calcium concentration was observed in fed sea bream with higher calcium levels in anterior and mid intestine (Figure 2) when compared to unfed fish.

CaSR expression reflected intestinal fluid calcium concentrations in the sea bream (Figure 2).

No effect was observed in plasma calcium between fed and unfed fish with 3.44 *±* 0.12 mmol·L^−1^ and 3.45 *±* 0.09 mmol·L^−1^, respectively.

### 2.3. HCO_3_^−^ Secretion

Basal intestinal secretion of HCO_3_^−^ using conditions similar to previous studies was 504 *±* 8.02 nmol·h^−1^·cm^−2^ which is in agreement with our previous work [9,29,47,48].

**Figure 3:**
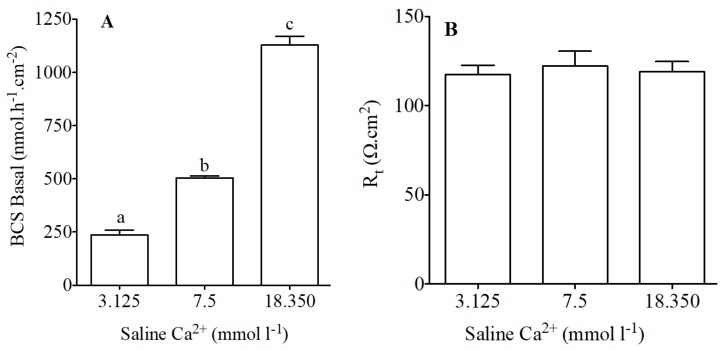
HCO_3_^−^ secretion (nmol·h^−1^·cm^−2^) as measured in Ussing chambers by pH-Stat (**A**) and tissue resistance (Rt, Ω·cm^2^); (**B**) in preparations from the anterior intestine of juvenile sea bream with an apical saline 3.125, 7.5 and 18.350 mmol·L^−1^ Ca^2+^. Each bar represents the mean ± SEM (*n* = 4–6). Different lowercase letters indicate significant differences (*p* < 0.05, one-way ANOVA, followed by the Bonferroni post hoc test).

Modified concentration of calcium in apical saline resulted in significant increases or decreases in HCO_3_^−^ secretion in the anterior intestine (Figure 3A). HCO_3_^−^ secretion in significantly increased when increased concentrations of calcium were used in apical saline (Figure 3A). Epithelial resistance did not change in response to apical calcium manipulation (Figure 3B).

**Figure 4:**
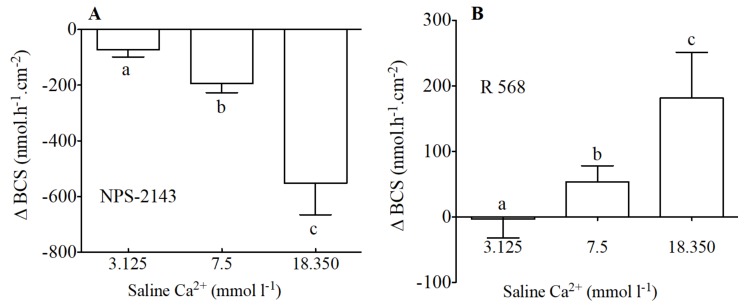
Variation of HCO_3_^−^ secretion (Δ nmol·h^−1^·cm^−2^) as measured in Ussing Chambers by pH-Stat in preparations from the anterior intestine of juvenile sea bream with an apical saline 3.125, 7.5 and 18.350 mmol·L^−1^ Ca^2+^ in response to apical addition of 100 μM of NPS-2143 (**A**) and 100 μM of R 568 (**B**). Each bar represents the mean ± SEM (*n* = 5–7). Different lowercase letters indicate significant differences (*p* < 0.05, one-way ANOVA, followed by the Bonferroni’s Multiple Comparison Test).

In addition to modification of calcium levels, NPS-2143 (Figure 4A) the allosteric antagonist of the CaSR, caused a significant decrease of HCO_3_^−^ secretion. The inhibitory effect of NPS-2143 was significantly higher when calcium levels were higher. Thus, apical NPS-2143 (100 μM) resulted in decreases of −72, −192 and −552 nmol·h^−1^·cm^−2^ in apical salines with 3.125, 7.5 and 18.350 nmol·L^−1^ Ca^2+^ respectively.

The opposite effect was observed in response to apical addition of R 568 an allosteric agonist of the CaSR (Figure 4B). R 568—sensitive HCO_3_^−^ secretion in the anterior intestine of sea bream increased in the presence of increasing levels of calcium in the apical saline. Thus, apical R 568 (100 μM) resulted in increases of −3, 53 and 181 nmol·h^−1^·cm^−2^ in apical salines with 3.125, 7.5 and 18.350 nmol·L^−1^ Ca^2+^ respectively.

### 2.4. Gravimetric Water Absorption

**Figure 5:**
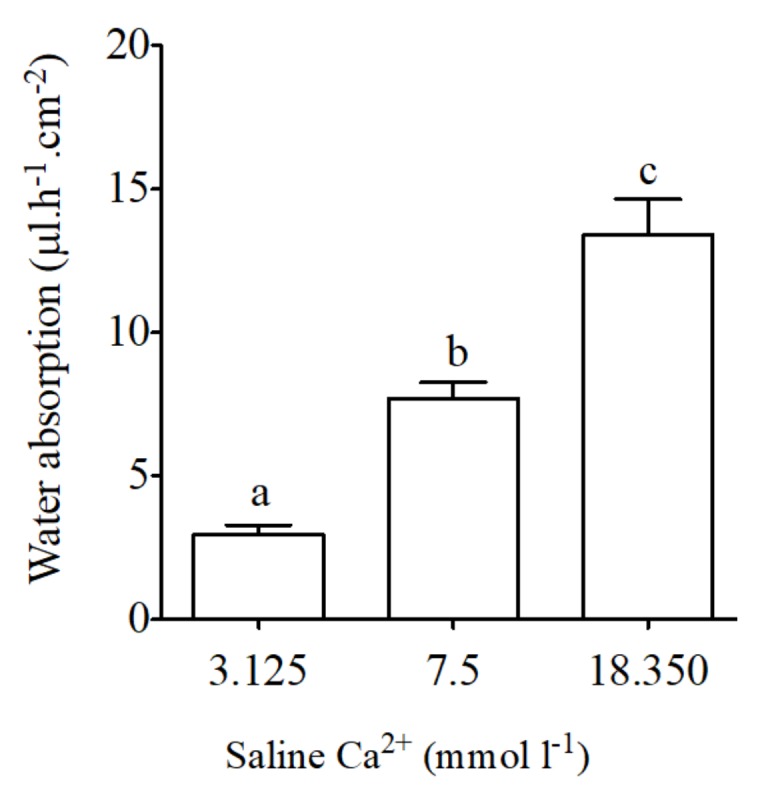
Water absorption (μL·h^−1^·cm^−2^) as measured in intestinal sac preparations in vivo-like conditions in the anterior intestine of the sea bream in response to different concentration of calcium in apical saline (3.125, 7.5 and 18.350 mmol·L^−1^ Ca^2+^ in). Each bar represents the mean ± SEM (*n* = 5–11). Different lowercase letters indicate significant differences (*p* < 0.05, one-way ANOVA, followed by the Bonferroni’s Multiple Comparison Test).

Basal water absorption using intestinal sacs from the anterior intestine of the sea bream was 7.7 *±* 0.6 μL·h^−1^·cm^−2^. Significant reductions in intestinal water absorption occurred in response to apical saline with low calcium concentrations (3.125 mmol·L^−1^ Ca^2+^, Figure 5). The opposite effect was observed in intestinal sacs in response to apical saline with high calcium concentrations (18.350 mmol·L^−1^ Ca^2+^, Figure 5).

**Figure 6:**
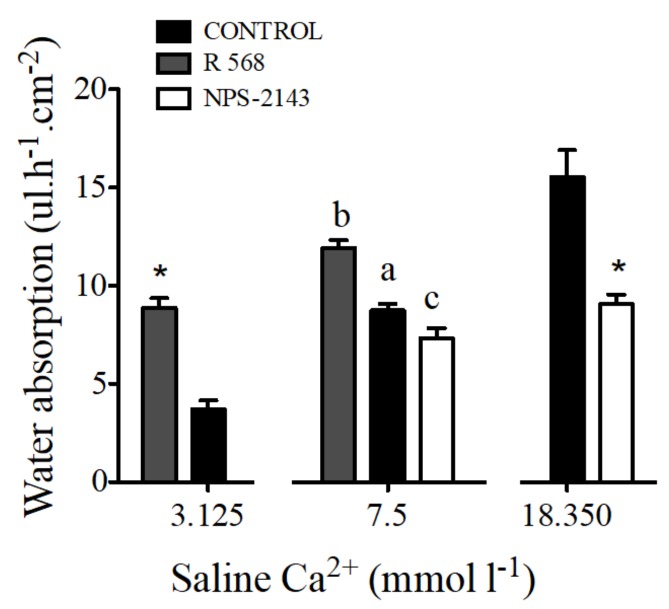
Water absorption (μL·h^−1^·cm^−2^) in response to R 568 (100 μM) or NPS-2143 (100 μM) in sea bream anterior intestine sacs with different working concentrations of apical calcium. Results are shown as mean ± SEM (*n* = 5–17). Asterisks represent significant differences in relation to the matching controls (*p* < 0.05, Student’s *t*-test); and different letters show significantly differences (*p* < 0.05, one-way ANOVA, followed by the Bonferroni’s Multiple Comparison Test).

The addition of 100 μM of NPS-2143 to luminal saline caused a decrease of water absorption by sac preparations from the anterior intestine. The opposite effect was observed after addition of 100 μM R 568 (Figure 6).

Water absorption in intestinal sacs of the anterior intestine of the sea bream increased in response to R 568 (Figure 6). A similar effect of both calcimimetics was observed in preparations with apical salines prepared with 3.125 and 18.350 mmol·L^−1^ Ca^2+^.

## 3. Discussion

The present study establishes a role for the calcium sensing receptor in the regulation of HCO_3_^−^ secretion and water absorption in the intestine of marine fish. The process of HCO_3_^−^ secretion is sensitive to physiological concentrations of calcium in saline media and it is affected by known agonists and antagonists of the CaSR.

There are recent studies that suggest a relationship between calcium transport and HCO_3_^−^ secretion in the intestine of marine fish in terms of regulation by endocrine factors or environmental effects [5,10,23,35,49,50]. The single common factor to all studies above cited is calcium. Here we hypothesised that calcium, via a CaSR, known to be expressed in sea bream intestine [51] could act as a direct regulator of intestinal HCO_3_^−^ secretion.

Flanagan et al. [51] described the presence of the CaSR in the intestine of sea bream. Here we qualified that study and confirmed the results by qPCR, showing that the CaSR is expressed along the whole intestinal tract of the sea bream. Additionally, we showed higher expression levels in the anterior intestine than in the mid intestine or the rectum. The presence of CaSR was also identified in the intestine of other fish species such as tilapia, *Oreochromis mossambicus* [52], winter flounder, *Pleuronectes americanus*, Atlantic salmon *Salmo salar* [53] and rainbow trout *Oncorhynchus mykiss* [32]. However, there is not such detailed study and our work reveals a specific pattern of expression in the intestine. Moreover, we demonstrated a feeding regulation of the CaSR expression in the intestine of sea bream, with significant expression increases 6 h after feeding, when the food progresses along the intestine. In addition to regulation of the CaSR expression we observed higher calcium in the intestinal fluid, reaching levels of ~18 mmol·L^−1^ after a single meal compared to control values of 7 to 8 mmol·L^−1^. This compares well with studies in toadfish [27] where an impact of feeding on osmoregulation was shown. Therefore, considering our starting hypothesis for a link between the CaSR and HCO_3_^−^ secretion we tested the effect of calcium alone, and our results suggest that HCO_3_^−^ secretion was associated with calcium availability.

The calcimimetics are pharmaceutical compounds that regulate (activate/block) the CaSR [32,44,45,46]. The calcimimetics that bind the CaSR as known agonists or antagonists bind to a site that is distinct from the physiological ligand and function as allosteric modulators of the CaSR, that amplify the sensitivity of the CaSR to serum calcium [54,55]. Here, we demonstrated that the calcimimetic NPS-2143 blocks the action of the sea bream CaSR and decreased the HCO_3_^−^ secretion. In contrast, the same dose of R 563 that activates the CaSR results in an increased HCO_3_^−^ secretion.

The involvement of the CaSR in the regulation of intestinal HCO_3_^−^ secretion is substantiated by the responsiveness to calcium and calcimimetic regulators. The addition of NPS-2143 to the apical side of in vitro preparations resulted in a reduced HCO_3_^−^ secretion in different conditions. When apical saline with low or high calcium concentration was used, the effect was in the same direction, and HCO_3_^−^ secretion was lower in all preparations. There are not similar studies in the literature linking CaSR to intestinal HCO_3_^−^ secretion. However, the usefulness of calcimimetics to evoke predictable effects via the CaSR in sea bream intestine is in agreement with previous studies in mammals, where NPS-2143 was shown to block Ca^2+^ receptor activity in rats [56]. On the other hand, the compound R 568 has the opposite effect in HCO_3_^−^ secretion in the sea bream intestine, increasing the basal values of secretion. A previous study in flounder (*Platichthys flesus*), suggests that the responses to R 568 administration results from calcimimetic induced increases in plasma STC-1 levels [57] via the CaSR. 

Our study suggests an additional role of Ca^2+^ in intestinal HCO_3_^–^ secretion, as the availability of calcium was directly associated with HCO_3_^−^ secretion in anterior intestine of the sea bream [29]. In keeping with this idea a previous study [28] of our group has shown a regulatory role for the CaSR in acid secretion in the stomach of the sea bream. Together with the regulatory role of the CaSR here demonstrated, for the process of HCO_3_^−^ secretion in the anterior intestine we might suggest a fundamental role for CaSR in the regulation of gastrointestinal re-adjustment to the feeding process. On the other hand, when salinity dependent HCO_3_^−^ secretion in the intestine of the sea bream, is considered [29], a parallelism exists with the calcium concentration in the intestinal lumen. In marine fish, the CaSR is essential for regulation of Ca^2+^ homeostasis [32,36,41,42,57].

How CaSR brings about the regulatory actions here demonstrated in bicarbonate secretion and water absorption remains to be demonstrated. However, previous studies of our group [5,9,10,29,47,48] have shown a regulatory role of endocrine factors in intestinal bulk water absorption such as PTHrP, but also of the regulation of HCO_3_^−^ secretion in the intestine of the sea bream such as prolactin or stanniocalcin. Especially interesting in the current context is the contrasting regulatory role by transmembrane and soluble adenylyl cyclase stimulation observed in water absorption and HCO_3_^−^ secretion in the intestine of sea bream [47], as it has been previously shown that agonists of the CaSR down-regulate cAMP accumulation [45]. These endocrine evidences support that HCO_3_^−^ secretion in the intestine of the sea bream is the link between carbonate precipitate formation and water absorption. But limited functional information exists on the relationship between calcium, regulation of HCO_3_^−^ secretion and water absorption. The mechanism behind CaSR signalling and bicarbonate secretion remains poorly understood in most epithelia. In fact the status of knowledge on the role of CaSR in epithelial bicarbonate secretion in fish is very limited. Previous studies in mouse and human have summarizes the role of calcium signalling in epithelia bicarbonate secretion and suggest that cytosolic calcium signalling can increase bicarbonate secretion by regulating membrane transport [58,59]. These evidences revealed that the cAMP-induced mechanisms in epithelial cells by the synergism between calcium and cAMP signals [58,59]. Our study reveals that calcium is critical to modulate bicarbonate secretion. Based in our previous work we suggest a link between the calcium sensing receptor and the soluble adenylyl cyclase that has been demonstrated to evoke increases in HCO_3_^−^ secretion in the sea bream [45] and promote NaCl and water absorption in toadfish intestine [60].

Previous studies have suggested that the mechanism of activation/inactivation of CaSR respond to changes in environmental Ca^2+^ levels [36]. In addition, our results suggest that the CaSR has a key role to fish calcium regulation, since the response to calcimimetics indicates that the CaSR functions as a sensing mechanism to modulate the “grand” process of HCO_3_^−^ secretion/water absorption/precipitate formation. Taken as a whole our results point to the idea that luminal calcium levels in the intestine may act as limiting or regulatory factors for intestinal precipitate formation. It appears that drinking may also be connected at the early stage of this complex physiological role mediated by calcium as shown in previous work of our group [34]. However, this suggestion requires future experimental demonstration. 

In conclusion, this study provides evidence to show that calcium availability to the intestinal lumen is directly associated with the regulation of HCO_3_^−^ secretion and water absorption in the intestine of the sea bream. This hypothesis is substantiated by the sensitivity of the intestine of the sea bream to known calcimimetics that activate or block the CaSR, at similar or different levels of fluid calcium.

## 4. Materials and Methods

### 4.1. Chemicals

All chemicals were of the highest grade and obtained from Sigma-Aldrich (Madrid, Spain). NPS-2143 hydrochloride a selective calcium-sensing receptor antagonist [44,54] was prepared in DMSO (dimethyl sulfoxide) as a 100 mM stock and was added to the apical side at final concentrations to achieve 100 μM. R 568 an allosteric agonist, which promotes CaSR stability [44,54] was prepared in DMSO as a 100 mM stock and was added to the apical side to achieve final concentration of 100 μM.

### 4.2. Animal Maintenance and Experimental Conditions

Sea bream (*Sparus aurata* L.) juveniles were obtained from commercial sources (CUPIMAR SA, Cadiz, Spain). Fish were quarantined for 60 days in Ramalhete Marine Station (CCMAR, Universidade do Algarve, Faro, Portugal) in 1000 L tanks in open-seawater circuits under natural conditions of water temperature (18–20 °C), photoperiod and salinity 37 ppt at a density of <5 kg·m^−3^ and fed twice daily with a commercial sea bream diet (Sorgal, Ovar, Portugal) containing 2.04 mg·g^−1^ of Ca^2+^ (calcium) and 1.47 mg·g^−1^ of PO_4_^3−^ (phosphate).

All animal manipulations were carried out in compliance with the Guidelines of the European Union Council (86/609/EU) and Portuguese legislation for the use of laboratory animals. All protocols were performed under a Group C license from the Direcção-Geral de Veterinária, Ministerio da Agricultura, do Desenvolvimento Rural e das Pescas, Portugal.

### 4.3. Food, Plasma and Fluid Calcium

In order to asses the effect of feeding in calcium and CaSR expression fish were either food deprived for 36 h to guarantee the absence of food from the intestinal tract. Another group was feed and sampled 6 h after feeding. This period was established to ensure the presence of various degrees of ongoing digestion along the intestine.

Fish (350–450 g body weight) were captured and anesthetized with 2-phenoxyethanol (1:10,000 *v*/*v*, Sigma, Madrid, Spain). Plasma was obtained by centrifugation of whole blood (10,000 rpm for 5 min), and stored at −20 °C for later analysis. After decapitation the intestinal fluid of individual fish was collected from the excised intestinal tract clamped (from pyloric caeca to anal sphincter) with two mosquito forceps, and emptied into pre-weighed vials and centrifuged (12,000× *g*, 5 min) to separate fluid from precipitate. To quantify calcium content in food, a known amount of feed (300–500 mg) was digested with 10 volumes of nitric acid for 48 h at room temperature and neutralized with 1 M NaOH and diluted as necessary with double-distilled water (ddWater).

Calcium content in food, in plasma and in intestinal fluid was measured by a colorimetric assay, using commercial kits (Spinreact, Reactivos Spinreact, SA, Girona, Spain), following the manufacturer’s instructions using a microplate reader Biorad Bench-mark (Bio-rad, Hercules, CA, USA).

### 4.4. Calcium-Sensing Receptor (CaSR) Expression

Intestinal samples were collected from individual fish, stored in RNA Later at 4 °C (Sigma–Aldrich) until utilized for RNA extraction within 2 weeks. Tissue from three sections of intestine was collected: (1) the anterior intestine, corresponding to 3–4 cm caudal to the point of insertion of the pyloric caeca; (2) Mid intestine, corresponding to the part between anterior intestine and rectum and (3) the rectum, which corresponds to a section of distal intestine, 2–3 cm in length, delimited by the anal and the posterior/rectal sphincters.

Total RNA was extracted from samples of the anterior intestine, mid and rectum with Total RNA Kit I (E.Z.N.A., Omega Bio-tek, Norcross, GA, USA) and the quantity and quality of RNA assessed (Nanodrop 1000, Thermo Scientific, Waltham, MA, USA). Prior to cDNA synthesis, RNA was treated with DNase using a TURBO DNA-free kit (Ambion by Life technologies, Invitrogen, Thermo Fisher Scientific, Waltham, MA, USA) following the manufacturer’s instructions. Reverse transcription of RNA into cDNA was carried out using the RevertAid First Strand cDNA Synthesis Kit (Thermo Fisher Scientific, Waltham, MA, USA) with 500 ng of total RNA in a reaction volume of 20 μL.

The cDNA sequences of CaSR were extracted from the EST collection database at the National Center of Biotechnology (NCBI, http://blast.ncbi.nlm.nih.gov/) using TBLASTn queries of known protein or deduced protein sequences from other fish species. Extracted sequences were compared by multisequence alignment using Clustal X to establish their identity [61]. Primer pairs were designed using the software Primer3 (version 0.4.0) (http://frodo.wi.mit.edu/) running under the EBioX (http://www.ebioinformatics.org/) interface for Macintosh. Table 1 shows primer sequences, amplicon sizes and NCBI accession numbers of the target sequences. Amplicon identities were confirmed by sequencing (CCMar).

Real-time qPCR amplifications were performed in duplicate in a final volume of 10 μL with 5 μL PerfeCTa FastMix II (Quantabio, Waltham, MA, USA) as the reporter dye, around 20 ng cDNA, and 0.3 μM of each forward and reverse primers (see Table 1). Amplifications were performed in 96-well plates using the CFX96 Touch Real-Time PCR detection system (Bio-Rad, Hercules, CA, USA) with the following protocol: denaturation and enzyme activation step at 95 °C for 2 min, followed by 40 cycles of 95 °C for 15 s, and primer pair specific annealing temperature for 10 s. After the amplification phase, a temperature determining dissociation step was carried out at 65 °C for 15 s, and 95 °C for 15 s. For normalization of cDNA loading, all samples were run in parallel using 18S ribosomal RNA (18S) To estimate amplification efficiencies, a standard curve was generated for each primer pair from 10-fold serial dilutions (from 1 ng to 0.0001 pg) of pooled first-strand cDNA template from all samples. In parallel a similar standard dilution was generated from the cloned genes of interest to confirm efficiencies and amplification of single products. Standard curves represented the cycle threshold value as a function of the logarithm of the number of copies generated, defined arbitrarily as one copy for the most diluted standard. All calibration curves exhibited correlation coefficients *R*^2^ > 0.98, and the corresponding real-time PCR efficiencies were >99%. Relative gene quantification was performed using the ΔΔ*C*t method [62].

### 4.5. Intestinal HCO_3_^−^ Secretion

All in vitro experiments were performed in unfed fish to avoid the confounding effects of feeding. Therefore, during the experiments feeding was withheld for 36 h before sample collection to guarantee the absence of undigested food in the intestine.

Fish (350–450 g body weight) were captured and anesthetized with 2-phenoxyethanol (1:10,000 *v*/*v*, Sigma, Madrid, Spain). After decapitation segments of anterior intestine were excised, mounted on tissue holders (P2413, 0.71 cm^2^, Physiologic Instruments, San Diego, CA, USA) and positioned between two half-chambers (P2400, Physiologic Instruments) containing 1.5 mL of basolateral and apical saline. The composition of these salines (see Table 2) simulated in vivo conditions for the sea bream as was previously described [5,29,47,48]: the basolateral saline was gassed with 0.3% CO_2_ + 99.7% O_2_ and the apical saline gassed with 100% O_2_ and pH maintained at 7.800 in apical saline during the experiments by pH-Stat.

Osmolality 340 mOsm·kg^−1^ adjusted with mannitol and pH was 7.800 and maintained constant with a gas mixture of 0.3% CO_2_ + 99.7% O_2_ in basolateral saline and by pH-Stat in apical saline.

Osmolality of all salines was adjusted to 340 mOsm·kg^−1^ with mannitol (Vapro 5520 Osmometer Wescor, South Logan, UT, USA). The temperature was maintained at 22 °C throughout all experiments. All bioelectrical variables were monitored by means of Ag/AgCl electrodes (with tip asymmetry <1 mV) connected to either side of the Ussing chamber with 3 mm-bore agar bridges (3 mol·L^−1^ KCl in 3% agar). Transepithelial electrical potential (TEP, mV) was monitored by clamping of epithelia to 0 μA cm^−2^. Epithelial resistance (Rt, Ω·cm^2^) was manually calculated (Ohm’s law) using the voltage deflections induced by a 10 μA·cm^−2^ bilateral pulse of 2 s every minute. Current injections were performed by means of VCC 600 amplifiers (Physiologic Instruments, San Diego, CA, USA). For pH-Stat control, a pH electrode (PHC 4000-8, Radiometer, Copenhagen, Denmark) and a microburette tip were immersed in the luminal saline and connected to a pH-Stat system (TIM 854, Radiometer, Copenhagen, Denmark). To allow pulsing (for Rt calculation) during pH measurements, the amplifier was grounded to the titration unit. The configuration of amplifier/pH-Stat system used in this study is similar to that first established for the characterization of HCO_3_^−^ secretion in the intestine of the Gulf toadfish [49,63] and in sea bream [9,29,47,48]. Measurement of HCO_3_^−^ secretion was performed on luminal salines at physiological pH 7.800 during all experiments. The volume of the acid titrant (2.5 mmol·L^–1^ HCl) was recorded and the amount of HCO_3_^−^ secreted (nmol·h^–1^·cm^–2^) was calculated from the volume of titrant added, the concentration of titrant and the tissue surface area (cm^2^) and normalized to time (h). Experiments were only carried out with a tissue sample if the voltage and HCO_3_^−^ secretion were stable over 1 h. These experiments were performed with control preparations and experimental preparations in the presence of apical modified calcium or added calcimimetics (100 μM) R 568 and NPS-2143 respectively, collected from the same fish for a more robust statistical approach.

### 4.6. Gravimetric Water Absorption

Intestinal water absorption in sea bream anterior intestine was measured as previously described [47,64,65] in asymmetric or in in vivo-like conditions. The whole intestinal tract was removed and placed in a Petri dish containing pre-gassed (0.3% CO_2_ + 99.7% O_2_) basolateral solution (see above). The lumen was flushed and cleaned, and the anterior intestine isolated. Intestinal sacs were prepared by sealing one of the ends with a Teflon tape ligature, filling it with apical physiological saline (asymmetric conditions, see above) before sealing the second end with a ligature. Once the gut sac was filled care was taken to remove gas bubbles and the sealed watertight preparation had an internal pressure of 15 cm of water in PE50 polythene tubing. The sacs were rested for 40 min in physiological solution gassed with 0.3% CO_2_ + 99.7% O_2_. For calculation of water absorption, the intestinal sacs were weighed to the nearest 0.1 mg, at 20 min intervals, over the duration of experiments (60–120 min). At the end of the experimental period, the sacs were opened, flattened and overlaid on millimetric paper to measure the surface area. The response to calcium or apical pharmacological NPS-2143 and R 568 (included in the saline) applied to the sac preparations was monitored during 60 to 120 min. Water absorption was expressed as μL·h^−1^·cm^−2^.

### 4.7. Statistical Analysis

Data are expressed as means ± SEM unless otherwise stated. Prior to statistical analysis, normality and homogeneity of variance were assessed. Differences between groups were tested using either Students *t*-test or as adequate using either one-way ANOVA or two-way ANOVA When ANOVA yielded significant differences, Bonferroni’s post-hoc test was used to identify significantly different groups. All statistical analyses were performed with Prism (version 5.0b) (GraphPad Software). Groups were considered significantly different at *p* < 0.05.

## Figures and Tables

**Table 1:** Primes used for q-PCR expression analysis. (Fw-forward; Rv-Reverse).

Gene	Primer	Sequence (5′ to 3′)	Tm (°C)	Amplicon (bp)	NCBI Accession No.
CaSR	Fw Rv	CAACCATTGCAGTTGTAGGAG AAGCGACTAGAGGAGGCGTAG	60	123	AJ289717.1
18S	Fw Rv	AACCAGACAAATCGCTCCAC CCTGCGGCTTAATTTGACTC	58	139	

**Table 2:** Composition of basolateral and apical salines used in vivo tissue experiments.

Constituents (mmol·L^−1^)	Basolateral	Apical	Apical High Ca^2+^	Apical Low Ca^2+^
NaCl	160	88	88	88
KCl	3	3	3	3
MgSO_4_	1	126.5	136	126.5
MgCl_2_		9.5	0	13.875
Na_2_HPO_4_		1	1	1
CaCl_2_	1.5	7.5	18.35	3.125
NaHCO_3_	5			
Glucose	5.5			
HEPES	5			
NaH_2_PO_4_	2			
